# Anti-Inflammatory, Antipyretic, and Analgesic Properties of *Potamogeton perfoliatus* Extract: In Vitro and In Vivo Study

**DOI:** 10.3390/molecules26164826

**Published:** 2021-08-10

**Authors:** Samar Rezq, Mona F. Mahmoud, Assem M. El-Shazly, Mohamed A. El Raey, Mansour Sobeh

**Affiliations:** 1 Department of Pharmacology and Toxicology, Faculty of Pharmacy, Zagazig University, Zagazig 44519, Egypt; samar_rezq@yahoo.com; 2 Department of Pharmacognosy, Faculty of Pharmacy, Zagazig University, Zagazig 44519, Egypt; assemels2002@yahoo.co.uk; 3 National Research Centre, Department of Phytochemistry and Plant Systematics, Pharmaceutical Division, Dokki, Cairo 12622, Egypt; elraiy@gmail.com; 4 AgroBioSciences Research, Mohammed VI Polytechnic University, Lot 660–Hay MoulayRachid, Ben-Guerir 43150, Morocco

**Keywords:** *Potamogeton perfoliatus*, inflammation, analgesia, antipyretic, antioxidant

## Abstract

Natural antioxidants, especially those of plant origins, have shown a plethora of biological activities with substantial economic value, as they can be extracted from agro-wastes and/or under exploited plant species. The perennial hydrophyte, *Potamogeton perfoliatus*, has been used traditionally to treat several health disorders; however, little is known about its biological and its medicinal effects. Here, we used an integrated in vitro and in vivo framework to examine the potential effect of *P. perfoliatus* on oxidative stress, nociception, inflammatory models, and brewer’s yeast-induced pyrexia in mice. Our results suggested a consistent in vitro inhibition of three enzymes, namely 5-lipoxygenase, cyclooxygenases 1 and 2 (COX-1 and COX-2), as well as a potent antioxidant effect. These results were confirmed in vivo where the studied extract attenuated carrageenan-induced paw edema, carrageenan-induced leukocyte migration into the peritoneal cavity by 25, 44 and 64% at 200, 400 and 600 mg/kg, p.o., respectively. Moreover, the extract decreased acetic acid-induced vascular permeability by 45% at 600 mg/kg, p.o., and chemical hyperalgesia in mice by 86% by 400 mg/kg, p.o., in acetic acid-induced writhing assay. The extract (400 mg/kg) showed a longer response latency at the 3 h time point (2.5 fold of the control) similar to the nalbuphine, the standard opioid analgesic. Additionally, pronounced antipyretic effects were observed at 600 mg/kg, comparable to paracetamol. Using LC-MS/MS, we identified 15 secondary metabolites that most likely contributed to the obtained biological activities. Altogether, our findings indicate that *P. perfoliatus* has anti-inflammatory, antioxidant, analgesic and antipyretic effects, thus supporting its traditional use and promoting its valorization as a potential candidate in treating oxidative stress-associated diseases.

## 1. Introduction

Multifactorial inflammatory reactions are the body’s first defense line against all pathogenic organisms, toxins, injuries and infections. However, an inadequate immune response might lead to several deleterious clinical consequences that boost the development of numerous inflammation-associated diseases. These include cancer, diabetes, asthma, obesity, rheumatoid arthritis and several neurodegenerative disorders [1,2,3].

The progression and the severity of such diseases are associated with the secretion of different inflammatory mediators and the production of excessive reactive oxygen species (ROS) as well. Additional acute and chronic pain, which is a common incident during various inflammation-associated diseases, worsens the health condition of the patients [1,2,3]. The harmful side effects of the nonsteroidal anti-inflammatory drugs used for managing inflammation and the associated pain are additional major concerns. Therefore, the development of safe, efficacious, multimechanistic agents that provide better medicines with lower costs is increasingly gaining importance. Plant-based medicine has been practiced since ancient times and has become a progressively appealing approach [1,4].

The pondweed family, Potamogetonaceae, comprises 110 species that are sectioned over six genera with *Potamogeton* as the largest genus [5]. Several plants from the genus have been utilized in folk medicine [5,6]. For instance, in China, *P. natans* L. is utilized in the treatment of the inflammation of the eye lining and in a combination with *P. perfoliatus* L. to cure some skin conditions. Infusions of the leaves of *P. natans* are utilized for stomach cramps and diarrhea, as well as for an antiscorbutic and for wound healing in Arabic medicine [7]. In Tibetan medicine, *P. berchtoldii* and *P. pusillus* are employed in the treatment of arthritis [7,8]. In addition, the broth of *P. distinctus* is used for the treatment of fish, meat, and alcohol food poisoning and the dried herb is mixed with soy flour and sugar to manage burns in Japanese medicine [7,8].

The redhead grass, *Potamogeton perfoliatus*, is a perennial hydrophyte that prevails in both standing and flowing freshwater habitats. It is commonly distributed worldwide and can be found on all continents except South America [9,10]. Eight flavonoids were reported from *P. perfoliatus*. These include apigenin and its 7-glucoside, luteolin and its 7-glucoside and 7-glucuronide, chrysoeriol and its 7-glucoside and isoorientin [11,12]. Palmitic (accounted for 30.97%), behenic, myristic, stearic, and lignoceric acids were also characterized using GC-MS from the aerial runners of plants grown in Russia. Rhamnose, glucose, galactose, mannose, fucose, and glucosamine, among other saccharides were identified from *P. perfoliatus* biomass [13]. Some studies highlighted several preliminary activities of *P. perfoliatus*, such as antibacterial, antifungal, antioxidant and antineoplastic effects [14,15]

Here, we investigated the antioxidant and anti-inflammatory effects of *Potamogeton perfoliatus* in vitro. We also explored the in vivo analgesic, anti-inflammatory and antipyretic effects. Furthermore, we characterized its secondary metabolites utilizing HPLC-PDA-MS/MS.

## 2. Material and Methods

### 2.1. Plant Material, Extraction and HPLC-PDA-ESI-MS/MS Analysis

The plant was collected from the Nile River, Zifta, Gharbia governorate, Egypt. The whole plant materials apart from the roots were washed with running tap water and air dried. A half kg of the dried material was extracted with hot boiling distilled water (5 L). The filtrate was reduced under vacuum to 300 mL. To clean up the extract, it was then re-extracted using ethanol several times until no precipitate was detected. The soluble aqueous ethanol extract was then evaporated to dryness to yield 38 g. HPLC-PDA-ESI-MS/MS profiling was performed as previously reported [16].

### 2.2. Cyclooxygenase and Lipoxygenase Inhibition Activities

The in vitro enzymatic activities were determined, utilizing enzyme immunoassay kits (Cayman Chemical, Ann Arbor, MI, USA) following the manufacturer’s instructions and the study by Adellall et al. [17].

### 2.3. Total Antioxidant Capacity (TAC) Assay

The antioxidant potential of the extract was evaluated by determining its TAC using a TAC ELISA kit (MBS726896, my BioSource, Inc., San Diego, CA, USA) in accordance with the manufacturer’s instructions and compared to the standard antioxidant, ascorbic acid as previously described Sobeh et al. [3].

### 2.4. In Vivo Studies

#### Experimental Animals

Male albino mice (8-week-old) weighing about 25 g and male Wistar rats (12-week-old) weighing about 200 g were utilized. After randomization, animals were kept in a controlled environment (temperature 22 ± 2 °C, humidity 60–70% and 12/12 h dark/light cycle) in individual polypropylene cages, fed ad libitum and given water. All experimental protocols were validated by the Institutional Ethics Committee of Zagazig University (protocol number, ZU-IACUC/3/F/115/2018).

### 2.5. Carrageenan-Induced Hind-Paw Edema Model

After randomization, 6 groups with 5 rats each were utilized in this experiment. They were two vehicle groups (10 mL/kg), diclofenac (20 mg/kg) and *P. perfoliatus* extract in three dose levels (200, 400, and 600 mg/kg). One hour later, edema in the rats’ right hind paw was induced by injecting 0.1 mL carrageenan solution (1% in 0.9% NaCl, freshly prepared), into the sub-plantar tissue in all groups but one vehicle group. The thickness of the edema (mm) was measured by caliper ruler in the dorsal-plantar axis before and after injecting the carrageenan solution, and at hourly intervals for 5 h, and finally at 24 h following the different treatments. The cumulative anti-inflammatory potential throughout the whole experiment period (0–24 h) was quantified by calculating the area under the changes in paw thickness-time curve (AUC_0–24_) [3].

### 2.6. Leukocytes Recruitment into Peritoneal Cavity in Mice

Male Swiss albino mice (*n* = 5–8/group), were subjected to orally gavage vehicle (1 mL/100 g), or *P. perfoliatus* extract in three dose levels (200, 400, and 600 mg/kg), 30 min before injection of carrageenan solution (0.1 mL, 500 µg/mice,) or sterile saline (0.1 mL). dexamethasone (2 mg/kg, p.o.) and diclofenac (20 mg/kg, p.o.) were used as reference drugs. Three hours later, the mice were euthanized, and each peritoneal cavity was washed with 3 mL of phosphate-buffered saline (PBS) containing 1 mM EDTA. The total leukocyte count was determined in each peritoneal cavity wash using a hemocytometer and presented as number of cells/mL [3].

### 2.7. Acetic Acid-Induced Vascular Permeability

To study the effect of the extract on the vascular phase of inflammation, acetic acid-induced vascular permeability was performed as described previously by Sobeh et al. [3]. Briefly, mice were treated with *P. perfoliatus* extract in three dose levels (200, 400, and 600 mg/kg, orally), dexamethasone (2 mg/kg), diclofenac (20 mg/kg), or vehicle. One hour later, Evans blue (0.25% solution in normal saline, 0.2 mL) was injected in the tail vein. Acetic acid (0.6% in normal saline, 1 mL/100 g) was then injected after 30 min in the peritoneal cavity of the treated mice. The normal control was injected with normal saline only. Mice were euthanized 30 min later by cervical dislocation, the abdominal cavities were washed with 3 mL saline solution and the collected washings were then centrifuged at 3000 rpm for 10 min. The vascular permeability is proportional to the absorbance of Evans blue dye in the supernatant, which was measured at 610 nm using a plate reader (Biotek, Winooski, VT, USA) [3].

### 2.8. Antinociceptive Activity by Hot Plate Test

To investigate the possible central analgesic activity of the studied extract, the hot plate test was performed according to Sobeh et al. [3]. Mice were randomly assigned into different groups (5 each) that received either *P. perfoliatus* extract (200 and 400 mg/kg, orally), the vehicle (10 mL/kg), or nalbuphine (10 mg/kg) as a reference drug. One hour later, each individual mouse was placed on a hot plate preheated at 55 ± 1 °C and observed for any response to the heat-induced nociceptive pain (licking the fore and hind paws, hind paw lifting, or jumping). The latency time until the animal showed the first signs of discomfort was recorded, before (baseline) and at 30 min, 1, 2, 3 and 4 h following the different treatments. The overall antinociceptive activity of the extract was estimated by calculating the area under the curve (AUC) for the latency responses at the stated time points using GraphPad Prism. The following formula was used to calculate the maximum possible analgesia (MPA) as the percent increase in overall latency time compared to untreated control: % increase in AUC = 100 × (AUC _treatment_ − AUC _control_)/AUC _control_.

### 2.9. Antinociceptive Activity by Acetic Acid-Induced Abdominal Writhing

The peripheral analgesic activity of the extract was evaluated by the acetic acid-induced writhing model in mice according to Sobeh et al. [3]. Animals were assigned to 4 groups (5–7 mice/each) that orally received either *P. perfoliatus* extract (200 and 400 mg/kg), diclofenac (20 mg/kg), or vehicle (1% Tween 80, 10 mL/kg), 1 h before the injection of 0.7% acetic acid (1 mL/100 g, i.p.). The number of writhes, manifested as extension of hind legs, constriction of abdomen, or turning of trunk was evaluated within 25 min. The reduction in the number of writhes as compared to the control group is indicative of the analgesic potential of the extract [3]. The percent inhibition of writhing was calculated using the following formula as previously reported [18]. % Analgesic activity = (mean writhing count (control-treatment)/mean writhing count (control)).

### 2.10. Antipyretic Activity in Brewer’s Yeast Induced Pyrexia in Mice

Pyrexia in mice was induced as previously reported by Liu et al. [19] with minor modifications. The animals’ initial rectum temperature was determined using a lubricated digital thermometer. Suspension of Brewer’s yeast was prepared in normal saline (30%) and injected subcutaneously behind the neck of the animal (1 mL/100 g). After 18 h, rectal temperatures of the mice were determined again (T_0_). Only animals that displayed rise in their rectal temperature (by at least 0.5 °C) were assigned to the experiments. Pyretic animals received *P. perfoliatus* extract (200, 400, and 600 mg/kg, orally), paracetamol (150 mg/kg), or vehicle. Rectal temperatures were measured at 30 min, 1, 2, 3 and 24 h following the different treatments [3].

### 2.11. Virtual Screening Studies

The molecular docking studies were done as previously described in Sobeh et al. [3].

### 2.12. Statistical Analysis

Statistical analysis was performed using GraphPad Prism, V6 (La Jolla, CA, USA). Parametric data were compared using one-way analysis of variance (one-way ANOVA) followed by Tukey post hoc test. Non-parametric data were compared using the Kruskal−Wallis one-way ANOVA followed by the Student–Newman–Keuls method for all pairwise multiple comparisons. Results were expressed as the mean and standard error and *p* < 0.05 values were considered statistically significant.

## 3. Results and Discussion

### 3.1. Phytochemical Profiling

**Figure 1:**
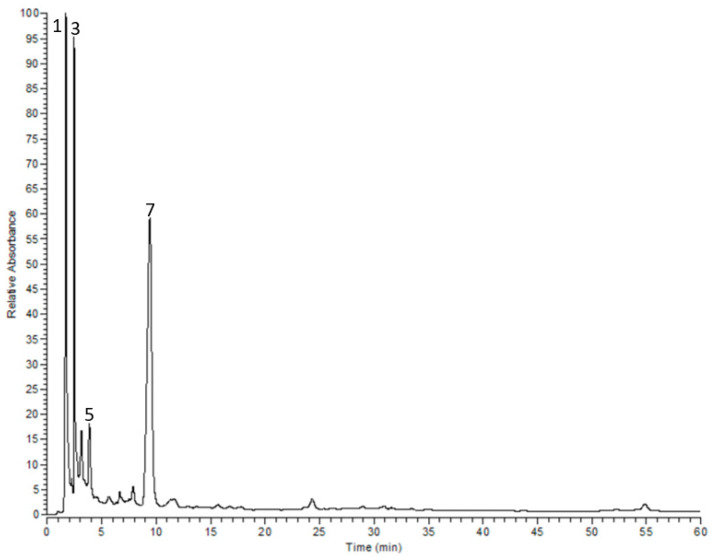
HPLC-UV chromatogram of *P. perfoliatus* extract.

Altogether, 15 secondary metabolites were annotated in *P. perfoliatus* extract using HPLC-PDA-MS/MS. Phenolic acids (mainly phloretic acid gallate, caffeic acid glucoside and *p*-hydroxybenzoic acid) dominated the extract. These compounds were annotated according to their molecular weights, fragmentation pattern and UV data. For instance, syringic acid (eluted at 2.96 min) was identified based on its [M − H]^−^ *m*/*z* at 197 and two fragment ions at *m*/*z* 153 and 179 [17]. A signal at 3.69 min exhibited an [M − H]^−^ *m*/*z* at 259 and a main fragment ion at *m*/*z* 179 [M − H − 80]; it was annotated as caffeic acid sulphate. Another signal at 6.74 min demonstrated a molecular ion peak [M − H]^−^ *m*/*z* at 441 and a major fragment at *m*/*z* 361 [M − H − 80] was assigned to catalpol sulphate. Quercetin, luteolin and apigenin derivatives were also detected in the extract, Figure 1 and Table 1.

### 3.2. Biological Activities

We explored the in vitro inhibition activities of the extract against LOX, COX-1, and COX-2. The extract showed more in vitro COX-2 selectivity than COX-1 with a selectivity index (SI = 289), which is comparable to the selective COX-2 inhibitor, celecoxib (SI = 271.66), Table 2. Moreover, the extract revealed lipoxygenase inhibitory activities with similar potency to the reference LOX inhibitor, zileuton, Table 2.

The natural phenolic acid and flavonoid contents of the extract may be responsible for the obtained inhibitory effects on the utilized enzymes. Similar activities were obtained from phenolic acid and flavonoid-rich extracts. For instance, leaf extracts from *Thymus algeriensis* and *Thymus fontanessi* from Algeria exhibited IC_50_ of 12.4 and 0.05 µg/mL on COX-1 and COX-2, respectively, and their activities were attributed to the presence of several phenolic acids, among them phloretic acid (identified in the tested extract) and apigenin, luteolin and quercetin glucosides (identified in the studied extract) [3]. Another study demonstrated that several fractions of pyroligneous acid obtained from palm kernel shell, rich in *p*-hydroxybenzoic acid (a major compound in our extract) exhibited substantial COX-1, COX-2 and LOX inhibitory activities [21]. Iridoid glucosides are widely distributed in aromatic and medicinal plants and act as prodrugs [22]. A sulphated derivative of the iridoid glycoside, catalpol, was annotated in the tested sample. Catalpol showed substantial antioxidant and anti-inflammatory activities as well as anticancer, antidiabetic and cardiovascular protective effects in experimental models [23].

Dual COX and LOX inhibition could provide a pharmacological advantage over single-enzyme inhibitors [24,25]. Plant extracts have shown solid potential due to their diverse composition that furnish several molecular targets compared to the nonsteroidal anti-inflammatory drugs (NSAIDs). The latter inhibits COX enzymes, but causes counter-regulatory mechanisms, such as LOX and leukotriene upregulation, which contribute to the major NSAID side effects [26]. Traditional NSAIDs also inhibit the cyclooxygenase pathway in a nonselective manner, causing allergic reactions and bronchospasm due to elevated leukotriene levels and gastric mucosal damage because of COX-1 inhibition. Selective COX-2 inhibitors have been linked to a higher risk of cardiovascular events [27]. As a result, novel anti-inflammatory agents with a dual action that prevent the release of both leukotrienes and prostaglandins are urgently needed.

Regarding the antioxidant potential, the tested extract showed a greater total antioxidant capacity (TAC) than that of ascorbic acid, the reference antioxidant compound, Table 2. Our results agree with those reported from other plant extracts rich in phenolics [3,4].

### 3.3. Effects of P. perfoliatus Extract on Carrageenan-Induced Paw Edema in Rats and Carrageenan-Induced Leukocyte Migration into the Peritoneal Cavity in Mice

**Figure 2:**
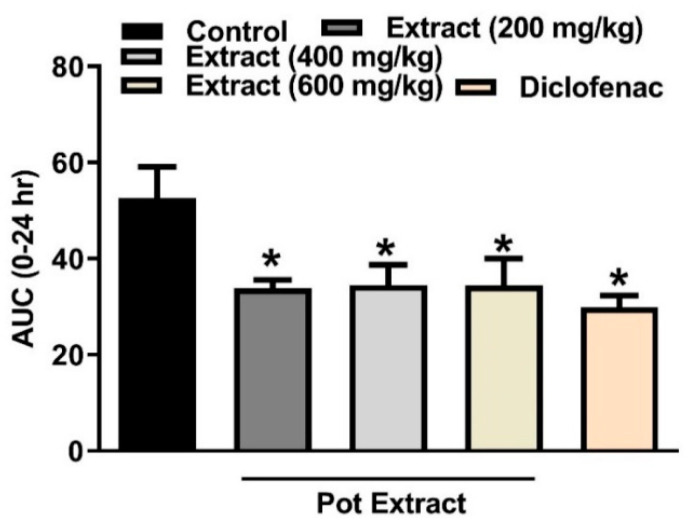
Results from *P. perfoliatus* extract (200, 400 and 600 mg/kg, p.o.) on carrageenan-induced paw edema in rats. The extract was administered 1 h prior to the carrageenan (1% suspension, 0.1 mL/rat) injection. Edema thickness (mm) was measured at one hour intervals for 5 h and at 24 h after carrageenan injection. The AUC_0–24_ results were expressed as mean ± S.E.M (*n* = 5). * *p* < 0.05 vs. control values.

Carrageenan-induced inflammation, first documented by Winter [28] is an acute, nonimmune, well-studied, and highly repeatable assay. Immediately after its subcutaneous injection, it induces edema, hyperalgesia, and erythema that are caused by proinflammatory agents, such as tachykinins, reactive oxygen, and nitrogen species, histamine, and bradykinin. Neutrophils can easily move to inflamed areas and produce pro-inflammatory reactive oxygen and other species [29]. Edema thickness can be reduced by drugs that target specific molecules in the inflammatory cascade. In the present study, the injection of carrageenan (0.1 mL, 1% in 0.9%, sub-plantar) increased paw thickness in rats when measured for 5 h at one hour-intervals for 24 h after injection. The tested extract, with its three dose levels 200, 400 and 600 mg/kg, p.o., decreased the edema thickness in rats, Figure 2. A similar pattern was observed from several plant extracts. We previously showed that a leaf extract from *Eugenia uniflora*, rich in flavonoids, decreased the AUC_0–24_ by 32% at 100 mg/kg, compared to 35 % by the current extract at a dose of 200 mg/kg [4]. *p*-Hydroxybenzoic acid (a major compound in our extract) significantly decreased the edema thickness at a dose of 20 mg/kg compared to the control group [30]. An aqueous fraction from the aerial parts of *Moricandia sinaica* Boiss., rich in flavonoids, among them quercetin and its glucosides, and phenolic acids such as phloretic acid gallate (identified in our extract), exhibited similar responses and was able to reduce the edema thickness in a dose-dependent fashion with its two doses (250 and 500 mg/kg) [31].

**Figure 3:**
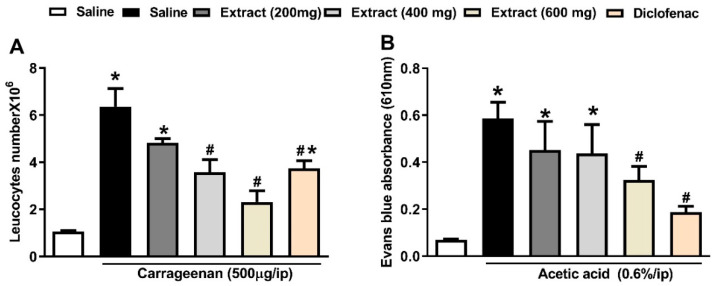
(**A**) Results from *P. perfoliatus* extract on carrageenan-induced leukocyte migration into the peritoneal cavity in mice (total number × 10^6^). Animals received the extract (200, 400 and 600 mg/kg, p.o.), diclofenac (20 mg/kg, p.o.) or vehicle 1 h before carrageenan injection (500 µg, i.p.). (**B**) Results from *P. perfoliatus* extract on acetic acid-induced vascular permeability. Mice received different treatments 1 h before acetic acid (0.6%, i.p.) and Evans blue dye injections. The amount of dye extravasation into the abdominal cavity was quantified and used as an indicator of inflammation degree. The results are shown as means ± SEM (*n* = 5–6). * *p* < 0.05 compared to saline group. ^#^ *p* < 0.05 compared to control (carrageenan or acetic acid only treated group).

In addition, the extract with its three tested doses (200, 400 and 600 mg/kg, p.o.) was able to decrease leukocyte migration by 25, 44 and 64%, respectively, in carrageenan-induced leukocyte migration into the peritoneal cavity in mice preinjected with carrageenan (500 μg/cavity, i.p., 0.1 mL), Figure 3A. The observed results agree with those reported by Hegazi et al. 2019 [32] in which a leaf extract from *Eugenia supra-axillaris* reduced leukocyte migration by 72% at the highest tested dose 400 mg/kg. An aqueous extract from *Ipomoea asarifolia*, along with its individual phenolic acids (chlorogenic acid and caffeic acid, annotated in our extract), also inhibited leukocyte migration [33]. Noteworthy, the effect of the middle dose of the studied extract (400 mg/kg) exhibited a comparable effect to the reference drug, diclofenac (20 mg/kg, p.o.), which decreased the number of leukocytes by 42%, Figure 3A.

### 3.4. Effects of P. perfoliatus Extract on Acetic Acid-Induced Vascular Permeability in Mice

It is well known that an intraperitoneal injection of acetic acid dramatically increases vascular permeability and enables Evans blue dye vascular leakage [34]. Endothelial cells in local capillary beds shrink after vasodilation in the initial stages of inflammation, resulting in gaps between the cells that significantly enhance vascular permeability. Fluid and cellular extravasation are facilitated by vascular permeability, resulting in localized edema in the inflammatory response [35]. Our results revealed that the extract, at a dose of 600 mg/kg, p.o., displayed an inhibition of 45% of vascular permeability. Notably, the reference drug, diclofenac, at a dose of 20 mg/kg, p.o achieved inhibition of 67% compared to control mice, Figure 3B.

### 3.5. Effects of P. perfoliatus Extract on Acetic Acid-Induced Writhing and Hot Plate Test in Mice

**Figure 4:**
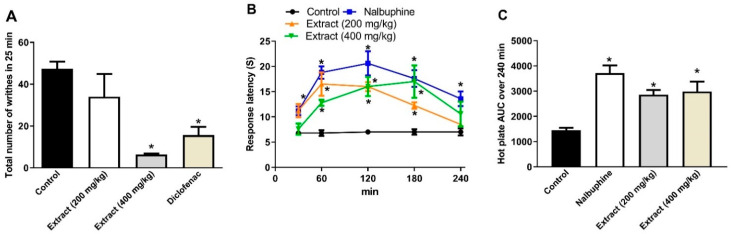
Analgesic activities of *P. perfoliatus* extract. (**A**) Acetic acid-induced writhing assay. Mice were treated either with the extract (200 and 400 mg/kg, p.o.) or diclofenac (20 mg/kg, p.o.) 1 h prior to the acetic acid (0.7%, 1 mL/100 g) injection. (**B**) Hot plate response latency (s) assay. (**C**) AUC of response latency shown in panel (**B**). The data results were quantified at one-hour intervals after administration of either the extract (200 and 400 mg/kg, p.o.) or nalbuphine (10 mg/kg, p.o.). Results are shown as mean ± S.E.M (*n* = 5–8). * *p* < 0.05 vs. control values.

With the goal of determining the possible peripheral and central antinociceptive effects of the extract, two separate analgesic testing procedures were used; the visceral pain model (acid-induced writhing) and the hot plate method as a central model. The acetic-acid writhing test is commonly used to investigate the analgesic effects of medicines on the peripheral nervous system. Injection of irritants such as acetic acid in mice causes a writhing response [36,37]. This is a chemical approach for inducing pain in the peripheral nervous system. We found that the extract, at a dose of 400 mg/kg dose, showed a robust analgesic activity by inhibiting the writhing response by 86%, Figure 4A. This action could be due to the suppression of COX enzyme and subsequent inhibition of the synthesis of the arachidonic acid metabolites [38]. It was reported that analgesia will be demonstrated by any drug that reduces the number of writhings, ideally by inhibiting prostaglandin synthesis, a peripheral mode of pain control [39]. Notably, this effect was superior to that obtained in mice pretreated with the reference standard, diclofenac (20 mg/kg, p.o.), which displayed 67% reduction of the control writhes, Figure 4A.

The hot plate method is commonly used for testing centrally acting analgesics. We observed that the two tested doses of the extract (200 and 400 mg/kg) showed a longer response latency when measured at 1, 2, and 3 h after administration. Notably, the highest tested dose (400 mg/kg) exhibited comparable effects to nalbuphine, the standard opioid analgesic, when assessed at later time points, Figure 4B. Additionally, the AUC estimated from Figure 4B indicated that the thermal latency against time was significantly increased at both tested doses by 96.22 and 104.94%, respectively, compared to the control value. The latter AUC values were not statistically different from that of nalbuphine, which showed an increase in AUC by 154.7%, Figure 4C. Taken together, the results from the hot plate assays suggested that the central analgesic activities of the tested extract might be attributed to the interactions with μ opioid receptors [40]. Other plants rich in phenolic acids and flavonoids offered similar activities [3,4,31,32].

### 3.6. Effects of P. perfoliatus Extract on Brewer’s Yeast-Induced Pyrexia in Mice

Antipyresis is debatable among caregivers, patients, clinicians, and scientists. A great number of caregivers think that fever is a disease, rather than a symptom, that may cause serious brain damage. There is some evidence that supports pyrogenic cytokines, such as interferons, interleukin (IL)-1, IL-6, and tumor necrosis factor (TNF), being responsible for fever, may have serious deleterious effects [41]. Fever reduction has numerous benefits including decreased insensible fluid loss, improved oral intake and the ability to rate the demeanor of the patients, as well as to decrease the febrile seizure risk [41]. NSAIDs interfere with the production of prostaglandin through nonselective inhibition of COX and therefore, downregulate COX enzyme expression that may cause severe side effects [41]. Novel selective COX-2 inhibitors are required to provide antipyretic effects with limited side effects from the inhibition of COX-1. As shown above, the tested extract showed selective COX-2 inhibition, so we further evaluated its antipyretic activities in Brewer’s yeast-induced pyrexia in mice. Brewer’s yeast, which is derived from the yeast Saccharomyces cerevisiae (*S. cerevisiae*), is widely used to induce pyrexia in rats in pharmacological studies and research [42]. It causes a significant rise in the synthesis of proinflammatory cytokines, key prostaglandin-producing enzymes, and transcription factors in the periphery and brain. We showed that a high dose of the extract (600 mg/kg) exhibited a pronounced antipyretic activity that started as early as one hour after the extract administration and continued until the next day. Notably, the latter effect was comparable to that observed with the standard antipyretic drug, paracetamol, Table 3. Comparable suppressive effects were reported from *Eugenia uniflora*, *Eugenia supra-auxillaris*, *Thymus algeriensis* and *Thymus fontanesii*, and *Moricandia siniaca* [3,4,31,32].

### 3.7. Virtual Screening Studies

Virtual screening via molecular docking was performed in order to gain some knowledge about how, at a molecular level, the main extract’s components are able to interfere with some molecular targets that are renowned for their important implication in the inflammation process. These include COX-1 and COX-2, which are the key enzymes utilized in the production of prostaglandins from the precursor arachidonic acid, 5-LOX that is responsible for the production of the proinflammatory leukotrienes via oxidation utilizing the same precursor, and finally the integral membrane protein FLAP, which is associated with the biosynthesis of leukotrienes as well.

**Figure 5:**
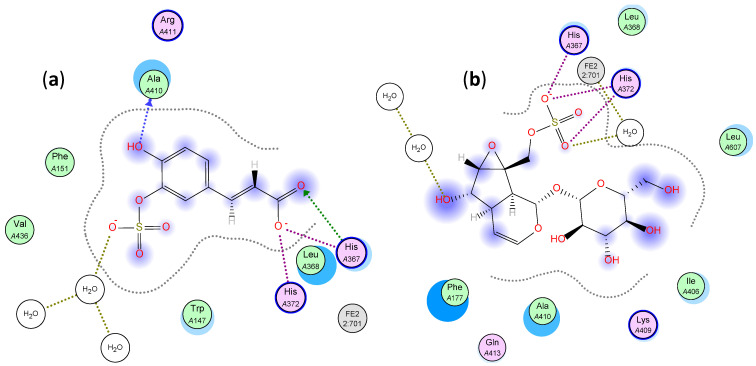
Two-dimensional interactions of (**a**) caffeic acid sulphate and (**b**) catalpol sulphate with 5-LOX.

We previously reported the interaction of caffeic acid glucoside, rosmarinic acid, luteolin glucoside with the key amino acids of COX-1 (pdb code: 5WBE), COX-2 (pdb code: 5IKR), 5-LOX (pdb code: 3V99), and 5-Lipoxygenase activating protein (FLAP, pdb code: 2Q7M). The three compounds exhibited low binding energy with the four tested molecular targets, Table 3 [3]. The other four annotated compounds were docked into the above-mentioned targets and their score functions are listed in Table 4. For 5-LOX, out of the docked compounds, caffeic acid sulphate showed the lowest binding energy and this was through two hydrogen bonds with Ala 410 and His 367 and three salt bridge interactions with His 367 and His 372, Figure 5a. Catalpol sulphate came as the second one with a binding energy of −16.92 kcal/mol and it bound through three salt bridge interactions with His 367 and His 372 and hydrogen bond through solvent with Fe^2+^, Figure 5b.

**Figure 6:**
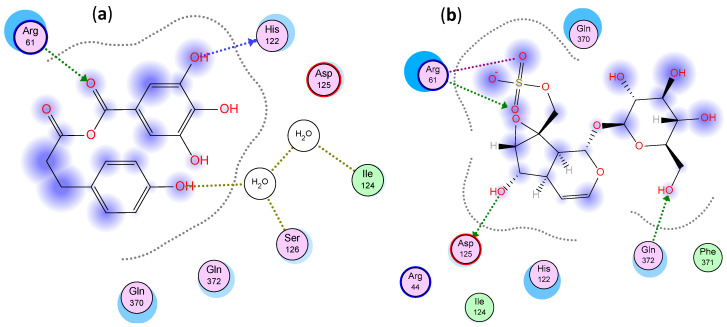
Two-dimensional interactions of (**a**) phloretic acid gallate and (**b**) catalpol sulphate with COX-2.

For COX-2, luteolin glucoside, rosmarinic acid and caffeic acid glucoside showed the lowest binding energy as shown before in Sobeh et al. [3]. Phloretic acid gallate and catalpol sulphate displayed pronounced binding energies as well, Table 4. Phloretic acid gallate bound through two hydrogen bonds with Arg 61 and His 122 and two other hydrogen bonds through solvent with Ile 124 and Ser 126 while catalpol sulphate bound through three hydrogen bonds with Asp 125, Gln 372 and Arg 61 and a salt bridge interaction with Arg 61, Figure 6. Noteworthy, all the docked compounds showed lower binding energies for COX-2 compared to COX-1, which confirms their higher selectivity towards COX-2, Table 4. It is worth mentioning that docking results were shown to be in agreement with the in vitro activities.

## 4. Conclusions

In conclusion, the chemical profiling of *P. perfoliatus* extract revealed the presence of 15 secondary metabolites belonging to phenolic acids and flavonoids. The obtained biological results suggest that *P. perfoliatus* extract has substantial antioxidant, anti-inflammatory, antinociceptive and antipyretic effects, and it can be considered as a potential candidate to ameliorate oxidative stress-associated diseases. These results can clarify its past and current usage; however, future studies are required to explore the detailed mechanism of the action of biomarker(s) from *P. perfoliatus* and its individual components.

## Figures and Tables

**Table 1:** Identified compounds from *P. perfoliatus* extract.

No.	Rt	[M − H]^−^	MS/MS	Proposed Compounds	References
1	1.25	317	125, 165	Phloretic acid gallate	[3]
2	1.58	341	161, 179	Caffeic acid glucoside	[3]
3	2.96	197	153, 179	Syringic acid	[20]
4	3.69	259	179	Caffeic acid sulphate	
5	4.11	163	119, 137	*p*-Coumaric acid	[20]
6	6.74	441	199, 361	Catalpol sulphate	
7	9.18	137	93, 137	*p*-Hydroxybenzoic acid	[20]
8	23.80	359	161, 197, 223	Rosmarinic acid	[3]
9	27.40	187	125, 169	Gallic acid derivative	
10	30.15	343	135, 179, 197	Coumaroyl syringic acid	
11	31.38	463	179, 301	Quercetin glucoside	[3]
12	34.92	447	179, 285	Luteolin glucoside	[3]
13	36.09	447	255, 301	Quercetin rhamnoside	[3]
14	37.74	563	269, 383, 443	Apigenin *C*-caffeoyl pentoside	
15	41.77	563	269, 443	Apigenin *C*-caffeoyl pentoside	

**Table 2:** In vitro inhibitory activities and total antioxidant capacity of *P. perfoliatus* extract.

Parameters	TAC	LOX	COX-1	COX-2	SI
	U/L	IC_50_ (µg/mL)	IC_50_ (µg/mL)		
*P. perfoliatus* extract	36.23 ± 2.10	3.00 ± 0.15	12.1 ± 0.31	0.042 ± 0.01	289.00
Ascorbic acid	28.27 ± 1.83	-	-	-	-
Diclofenac	-	2.83 ± 0.07	4.19 ± 0.18	0.87 ± 0.06	4.82
Celecoxib	-	-	16.30 ± 0.95	0.06 ± 0.01	271.66
Indomethacin	-	-	0.1 ± 0.01	0.82 ± 0.11	0.12
Zileuton	-	3.30 ± 0.06	-	-	-

Results are mean ± SE. SI is COX selectivity index which is defined as IC_50_ (COX-1)/IC_50_ (COX-2).

**Table 3:** Results from *P. perfoliatus* extract on Brewer’s yeast-induced pyrexia in mice.

Sample	Dose (mg/kg)	Rectal Temperature (°C)	Rectal Temperature Recorded Following Different Treatments (h)				
			0.5	1	2	3	24
Control	-	38.78 ± 0.26	38.88 ± 0.16	38.66 ± 0.19	38.86 ± 0.19	38.70 ± 0.20	38.34 ± 0.21
*P. perfoliatus* extract	200	37.94 ± 0.36	38.24 ± 0.13	38.84 ± 0.46	38.62 ± 0.21	38.28 ± 0.24	37.26 ± 0.63
	400	38.86 ± 0.33	39.10 ± 0.32	38.96 ± 0.27	38.52 ± 0.15	38.74 ± 0.16	37.84 ± 0.45
	600	38.56 ± 0.15	38.73 ± 0.14	37.40 ± 0.20 *	37.5 ± 0.4 *	37.30 ± 0.17 *	37.50 ± 0.24
Paracetamol	150	38.60 ± 0.18	38.14 ± 0.19	37.50 ± 0.31	37.12 ± 0.29 *	36.96 ± 0.24 *	36.50 ± 0.22 *

Results are shown as mean ± S.E.M (*n* = 5), * *p* < 0.05 vs. control values. 18 h after yeast injection.

**Table 4:** Results from docking of selected compounds from *P. perfoliatus* extract into the binding sites of 5-LOX, COX-1, COX-2, and FLAP.

Secondary Metabolite	Score Function (kcal/mol)			
	5-LOX	COX-1	COX-2	FLAP
Caffeic acid glucoside *	−12.90	−15.79	−18.14	−16.35
Rosmarinic acid *	−12.72	−16.20	−19.46	−15.09
Luteolin glucoside *	−18.46	−17.89	−24.48	−14.39
Phloretic acid gallate	−16.26	−10.43	−16.97	−8.14
Caffeic acid sulphate	−19.60	−10.68	−10.89	−8.06
p-Hydroxybenzoic acid	−12.72	−7.95	−10.09	−6.86
Catalpol sulphate	−16.92	−10.51	−10.91	−8.36

* Previously reported in Sobeh et al. [3].

## Data Availability

All data are included at the manuscript.

## Sample Availability

The extract is available from authors.
